# Antimicrobial activities evaluation and phytochemical screening of some selected medicinal plants: A possible alternative in the treatment of multidrug-resistant microbes

**DOI:** 10.1371/journal.pone.0249253

**Published:** 2021-03-26

**Authors:** Taye Kebede, Eshetu Gadisa, Abreham Tufa

**Affiliations:** 1 Department of Biomedical Sciences and Immunology, Natural Sciences College, Madda Walabu University, Bale-Robe, Ethiopia; 2 Department of Medical Laboratory Science, Menelik Medical and Health Science College, Kotebe Metropolitan University, Addis Ababa, Ethiopia; 3 Department of Biochemistry, Natural Sciences College, Madda Walabu University, Bale-Robe, Ethiopia; Nitte University, INDIA

## Abstract

**Background:**

Four out of five individuals rely on traditional medicine for their primary healthcare needs. Medicinal plants are endowed with diverse bioactive compounds to treat multidrug-resistant (MDR) microbes. So far, a less thorough examination has been made in this regard. This study aimed to evaluate antimicrobial activity and phytochemical screening of selected medicinal plants against MDR microbes.

**Methods:**

In vitro experimental study was carried out to evaluate antimicrobial effects and phytochemical screening of *Rumex abyssinicus*, *Cucumis pustulatus*, *Discopodium penninervium*, *Lippia adoensis*, *Euphorbia depauperata*, *Cirsium englerianum*, and *Polysphaeria aethiopica* against MDR bacteria and fungi. Aqueous and 80% methanolic extraction methods were employed for extraction. The susceptibility test, minimum inhibitory concentration, and minimum bactericidal or fungicidal concentration were measured using disc diffusion or broth micro-dilution as per the CLSI protocols.

**Result:**

The 80% methanolic extraction method was a preferred method to aqueous. The phytochemical constituents identified were alkaloids, flavonoids, saponins, phenolic, tannins, terpenoidss, and cardiac glycosides. The hydroalcoholic extract demonstrated an appreciable antimicrobial role against MDR microbes with an MIC value of 1.0–128.0μg/ml and 11-29mm inhibition zone (IZ) in diameter. Extracts obtained from *C*. *englerianum* and *E*. *depauperata* showed a significant IZ ranged of 26-29mm on MRSA and *Streptococcus pyogenes*. MDR *E*. *coli* and *K*. *pneumoniae* showed 12-25mm and 23-28mm IZ in diameter, respectively. *T*. *mentagraphytes* was susceptible to all tested extracts. Moreover, *S*. *pyogenes* and *K*. *pneumoniae* were found the most susceptible bacteria to *C*. *englerianum*. *Cirsium englerianum*, *L*. *adoensis*, *D*. *penninervium*, *and R*. *abyssinicus* demonstrated remarkable antifungal effect against *C*. *albicans* and *T*. *mentagrophytes*, while *R*. *abyssinicus* showed the leading antifungal effect with 32 to 64μg/ml MIC values.

**Conclusion:**

The plant extracts have shown appreciable antimicrobial activities comparable to the currently prescribed modern drugs tested. Accordingly, further studies on clinical efficacy trial, safety, toxicity and affordability analyses have to be instigated promptly, so as to head to the final step to synthesize precursor molecules for new effective antimicrobials.

## Introduction

Natural medicines have been used to boost health since the time of immemorial and the success of modern medical science largely depends on drugs originally obtained from natural resources. In the past, a large number of antimicrobial compounds were discovered from synthetic and natural products for the treatment and control of infectious agents [1]. However, only a few of them were reachable to the needy world’s market [2]. The emergence of multidrug-resistant bacteria has further compromised the accessibility and affordability of many currently prescribed antibiotics worldwide [3–5]. As a result, it reduces the effectiveness of the treatment regimens and increases morbidity, mortality, and health care costs [6–8]. According to the CDC report, each year in the United States, at least 2 million people acquire serious infections with bacteria that are resistant to one or more of the antibiotics used for the treatment of infections. The total economic cost of antibiotic resistance was estimated as high as $20 billion in direct healthcare and $35 billion dollars loss in productivity in a single year. The situation is further complicated in low-income countries by lack of effective surveillance systems, laboratory diagnostics, and access to appropriate antimicrobials in the face of financial limitations. If there were no successful efforts to intervene in terms of looking for new drugs, the number of deaths will rise to ten million and costs the world up to $100 trillion by the year 2050 [9–16]. To this effect, the search for an innovative antibiotic from natural products is ultimately an important segment of modern medicine to overcome the socio-economic and health impact caused by multidrug-resistant microbes [17].

The therapeutic agents derived from plants are justified by the emergence of diseases and the growth of scientific knowledge about herbal medicines as important alternatives or complementary treatment of diseases [18]. Many studies have shown that medicinal plants contain coumarins, flavonoids, phenolics, alkaloids, terpenoids, tannins, essential oils, lectin, polypeptides, and polyacetylenes [19–21]. These bioactive compounds are used as a starting point for antibiotics synthesis in order to treat infectious diseases [22]. The crude extract of *Polygonum persicaria*, *Rumex hastatus*, *Rumex dentatus*, *Rumex nepalensis*, *Polygonum plebejum*, and *Rheum australe* have antibacterial and antifungal activities, inhibit the growth of *C*. *frundii*, *E*. *coli*, *E*. *aerogenes*, and *S*. *aureus* [23]. The n-hexane extracts of *Calotropis gigantean* have no antibacterial and antifungal activities, against pathogenic microbes. But, its ethyl-acetate fraction has inhibitory effects on some bacteria and fungi except *T*. *rubrum* [24]. *Calotropis gigantean* crude extracts show promising antifungal activity against *Candida albicans*, *Aspergillus niger*, *Aspergillus ochraceus*, *Aspergillus ustus*, and *Rizopus oryzae* pathogenic fungi in Asia [21]. In another study, an ethanolic extract of *Plumbago zeylanica* root exhibit good antimicrobial activities against *V*. *cholerae*, *E*. *coli*, *P*. *aeruginosa*, *Curvularia lunata*, *Colletotrichum corchori*, and *Fusarium equiseti* [25]. The aqueous leaf extract of *Euphorbia hirta*, *Erythrophleum suaveolens*, and the methanolic leaf extract of *Thevetia peruviana* has an antibacterial effect against Extended Spectrum Beta-Lactamase (ESBL) producing bacteria such as *E*. *coli*, *Pseudomonas*, *K*. *pneumonia*, Methicillin-Resistant *Staphylococcus aureus* (MRSA), Salmonella and Proteus [26–28]. The few studies conducted on the aqueous and hydro-alcoholic extracts from different plants implicate antibacterial effect on multidrug-resistant bacteria including MRSA and ESBL producing bacteria [29, 30]. Moreover, some plants may exhibit broad-spectrum of antimicrobial effects, which possibly control the impairments associated with multidrug-resistant microbes [31].

Similarly, it has been pointed out that chemical synthesis and the search for natural products from living organisms (such as higher plants) are the major sources to look for a new bioactive compound to resolve human ailment due to pathogenic microbes, as more than 80% of the world’s population relies on traditional medicine for their primary healthcare needs [29]. However, until recently less than 1% of plants are characterized for their secondary metabolites, phytochemicals constituents, and pharmacologically active ingredient [30]. In this regard, traditional medicinal plants (TMP), are the most valuable source of new bioactive chemical entities due to their ecological biodiversity and diverse chemical endowment within each species [31]. Thus, *P*. *aethiopica*, *E*. *depauperata*, *C*. *englerianum*, *L*. *adoensis*, *C*. *pustulatus*, *D*. *penninervium*, and *R*. *abyssinicus* are TMP used by communities for the treatment of different disease caused by notorious infectious agents. Yet, there are limited detailed and thorough examinations of these plants for their potential role as antimicrobials and phytochemical entities as therapeutic agents for MDR bacteria and pathogenic fungi.

## Materials and methods

*In vitro*, an experimental study was carried out to evaluate the antimicrobial status and phytochemical screening of *P*. *aethiopica*, *E*. *depauperata*, *C*. *englerianum*, *L*. *adoensis*, *C*. *pustulatus*, *D*.*penninervium*, and *R*. *abyssinicus* against clinical isolate multidrug-resistant microbes and their respective reference strains.

### 2.1 Medicinal plants selection criteria

In this study, the selected medicinal plants were collected from Berbere district in Bale Zone, being guided by the local traditional healers and resident farmers who utilize these traditional medicines. Berbere district is situated between 06°33ʹ N and 06°75ʹ N and 039°95’ E and 040°29ʹ E. It is located about 526 km away from the Ethiopia capital city (Addis Ababa) in the southeast direction, in Bale Zone of Oromia Regional State. The district has 17 peasant associations, where the northern and southern parts are more of highlands and lowlands respectively. Out of the 17 peasant associations, the medicinal plants were collected from seven peasant associations of Berbere district based on the profundity of traditional knowledge generated during the pilot study interview; namely Burkitu (*Lippia adoensis*), Chekata (*Polysphaeria aethiopica*), Darasa (*Cucumis pustulatus*), Gabe Keka (*Discopodium penninervium*), Gora Heddo (*Rumex abyssinicus*), Harawa (*Cirsium englerianum*), and Sirrima (*Euphorbia depauperate*).

Berbere district is far away from the Zonal city (Bale-Robe) and gets less coverage of infrastructures (in terms of hospital, transport, electrical power supplies, and other social security rendering institutions). Until the conclusion of these fieldworks (plant collection and interviews), there is only one health center in the district and the residents are imposed to use medicinal plants for the treatment of skin diseases, diabetes, Urinary Tract Infections, hepatitis, Sexually Transmitted Diseases, cancer, hypertension, sexual impotence, and as contraceptives. Of which, *P*. *aethiopica*, *E*. *depauperata*, *C*. *englerianum*, *L*. *adoensis*, *C*. *pustulatus*, *D*. *penninervium*, and *R*. *abyssinicus* are used by healers for the treatment of bacterial infection such as eczema, gonorrhea, syphilis, pneumonia, scabies, skin infection, and superficial mycosis.

### 2.2 Plant collection

The leaf, bark, and root parts of seven plants, *P*. *aethiopica*, *E*. *depauperata*, *C*. *englerianum*, *L*. *adoensis*, *C*. *pustulatus*, *D*. *penninervium*, and *R*. *abyssinicus* were collected and screened for their phytochemical bioactive ingredients and evaluated for antimicrobial effects on common human pathogens (bacteria and fungi). The collected plant materials were handled with standard storage protocols and transported being wrapped by plastic sheets. Authentication of each plant sample was carried out and deposited at the National Herbarium, Department of Biology, College of Natural and Computational Sciences, Addis Ababa University (AAU), Addis Ababa, Ethiopia.

### 2.3 Preparation of plant extracts

The collected plant parts were washed thoroughly under running tap water and rinsed in distilled water, air-dried at room temperature under shade (23–27°C), and reduced to appropriate size. The powdered plant materials were packed in a plastic bag and kept until extraction. The powdered plant materials were weighed by sensitive digital weighing balance and a total of 1.2 g of powdered aerial were macerated with 80% methanol (250 g in 1500 mL) in an Erlenmeyer flask and also separately soaked in 500ml of distilled water for three days at room temperature (23–27°C). The extraction process was facilitated by occasional shaking. After three days of frequent agitation, the extract was separated from the marc using gauze and the resulting liquid was filtered using Whatman filter paper No. 3 (Whatman Ltd., England). The residue was re-macerated and repeated three times to exhaustively extract the plant material. The filtrates obtained from the successive maceration were concentrated under reduced pressure using a rotary evaporator (Hamato, Japan) followed by a hot air oven (Medit-Medizin Technik, Germany) set at 40°C. Extraction was repeated seven times and the filtrates of all portions were combined in one vessel. The organic solvent was removed by evaporation using a Rotary evaporator at 40°C. After the removal of the organic solvent, the aqueous residue was placed in a lyophilizer until non-polar solvents were removed and the extracts became dried. The extract was further concentrated to dryness by freeze-drying using a lyophilizer (Labfreez, China). After drying, the amount of dry extract obtained was harvested and the dried extract was transferred into airtight bottles, where the resulting dried mass was packed into a glass vial and stored in a desiccator over silica gel and stored in a refrigerator at −4°C until use. The weight of the dry extract was expressed as a percentage of the total mass of dry plant matter to determine the percentage yield [18, 31].

### 2.4 Sterility test of the plant extracts

Each extract of methanol and aqueous was tested for the growth of microbes. This was carried out by inoculating 0.5ml of each of them on sterile Mueller Hinton Agar and incubated at 37°C for 18–24 hrs. The fungal growth was tested on Sabouraud Dextrose Agar for 4 days being incubated at 25°C. The plates were observed for growth. The absence of growth in the extracts after incubation indicates sterility and evaluated for antimicrobial activity as indicated in CLSI guidelines [32].

### 2.5 Culture media

Nutrient Agar, TSY Broth, MacConkey, Muller Hinton Agar (MHA), Muller Hinton Broth (MHB), Blood Agar (BA), Mannitol Salt Agar (MSA), Chocolate Agar, and biochemical reagents for bacteria and Sabouraud Dextrose Agar (SDA) for fungi were obtained from the Department of Medical Microbiology, Immunology and Parasitology, Addis Ababa University and Tikur Anbessa Specialized Hospital (TASH) Bacteriology and Pharmacology Units.

### 2.6 Test microorganisms

The reference bacterial species; American type cell culture (ATCC) of *Escherichia coli* (ATCC25922), *Klebsiella pneumoniae* (ATCC700603), *Streptococcus pyogenes* (ATCC 19615), and *Staphylococcus aureus* (ATCC 25923) and their respective MDR strains, and *Trichophyton mentagrophytes* (ATCC 18747) and *Candida albicans* (ATCC 10535) were collected from Bacteriology Unit of the Microbiology Laboratory of TASH. All laboratory works were performed according to CLSI guidelines [33].

### 2.7 Modern antibiotics

The antimicrobial discs used for this research were the products of Liofilchem® Inc. (Liofilchem, Clinical and Industrial Microbiology Company) produced in 2017. The modern antibiotics discs employed were Ciprofloxacin (5μg/disc), Gentamycin (10μg/disc), Cephalothin (30μg/disc), Cefotaxime (5μg), Ceftazidime (10μg), Cefoxitin (30μg), Ceftriaxone (30μg), Amikacin (30μg), Cefuroxime (5μg), Ceftriaxone (30μg), Cloxacillin (30μg), and Augmentin (30μg) for the bacteria while the modern antifungal drug, Ketoconazole (20μg) was used for *C*. *albicans* and *T*. *mentagrophytes* as per the CLSI guideline protocols [32, 33].

### 2.8 Screening for multidrug-resistant bacteria

Multidrug-resistant gram-negative and gram-positive bacteria were isolated from three different samples (urine, throat swab, and blood). All bacterial cultures were first grown on 5% blood agar plates at 37°C for 18–24 hours before inoculation onto the MHA. Few colonies (3 to 5) of similar morphology of the respective bacteria were transferred with a sterile inoculating loop to a liquid medium until adequate growth of turbidity with McFarland in 0.5. Then the bacterial suspension was streaked onto MHA plates using a sterile swab in such a way as to ensure thorough coverage of the plates and a uniform thick lawn of growth following incubation. The susceptibilities of clinical isolates were tested by using the MHA impregnated with a range of antimicrobial agents. Dilutions of overnight broth cultures were inoculated onto antibiotic-containing plates to yield final inoculums of approximately 10^6^ CFU per spot according to CLSI protocol guidelines for Enterobacteriaceae. Selected multidrug-resistant, *K*. *pneumoniae*, and *E*. *coli* were screened for their resistance for more than two different classes of antibiotics following the disk diffusion method protocol (as in the CLSI guidelines) and WHO recommendations [33].

### 2.9 Determination of MIC and MBC values

After preliminary screening of plants for their antimicrobial activity, those which revealed potent antimicrobial effects were further tested to determine MIC and MBC against multidrug-resistant gram-negative, gram-positive, and Candida species. It was determined by the MHB broth micro-dilution method. Each of the 96-well microtiter plates was liquated with 50μl of MHB; while 100μl of MHB was added to the 10th well (sterile control). MHB with 5% DMSO added to the 9th well (growth control). About 50μl of each extract initially dissolved in 5% DMSO, which was dispensed into the first well. A serial two-fold dilution was performed by transferring 50μl of the suspension to the subsequent wells until the 8th well as per the protocol and procedure is given by CLSI guidelines and the modified protocol of Wiegand [32]. About 0.5 McFarland broth inoculum was diluted in the ratio of 1:100 and dispensed to 1 to 8th wells to attain the final inoculum size of 5 x 10^5^ CFU per ml [32–34].

Bacterial cell viability and MIC values were determined by turbidity test. The lowest concentration of the extract with clear suspension was considered as the MIC values. The lowest concentration of the extract in the post-incubation suspensions which did not harbor any bacterial growth upon spotting on MHA after overnight incubation at 37°C was considered as the MBC values. The method adopted for the fungus was the same as that of the bacteria. Instead of nutrient agar, Sabouraud dextrose agar was used. The inoculated medium was incubated at 25°C for C. *albicans* and *T*. *mentagrophytes*. The test was performed in triplicates alongside antibiotics ciprofloxacin (5μg) as a positive control at a concentration of 0.1mg/ml for bacteria. Ketoconazole was used as a positive control at a concentration of 0.3mg/ml for fungi [33, 35].

### 2.10 Phytochemical screening

Standard preliminary phytochemical qualitative analysis of the extract was carried out for the various plant constituents and screened for the presence or absence of biologically active compounds or secondary metabolites using standard procedures. As a result, the major phytochemical constituents identified were alkaloids, anthraquinones, cardiac glycosides, flavonoids, phenolic compounds, saponins, tannins, and terpenoids in 80% methanol extracts of each plant using modified standard procedures [19, 31, 36], as applied in one of the previous research work on combined antibacterial effect of essential oils [35].

#### Test for alkaloids

Around 200mg plant material was boiled in 10mL methanol and filtered. Then, 1% HCl was added followed by 6 drops of Dragendorff reagent. The brownish-red precipitate was taken as a piece of evidence for the presence of alkaloids. Towards the end, an alkaloids test was applied on few plant materials by diluting 2.5mg of the extract with 2.5ml of 1% HCl in a tube and boiled. Then, 1ml of the filtrate was added to 1ml of dilute ammonia. Finally, 1ml of chloroform (CHCl_3_) was added and shaken gently to reveal the alkaloid base. **Test for anthraquinones** (Borntrager’s Test): About 1mg of each extract was reacted with 2ml benzene, shaken properly, and filtered through Whatman’s no. 1 filter paper. Then, the filtrates were allowed to reacted with 2.5ml of 10% ammonia solution and shaken properly. The presence of pink, red, or violet color in ammonia solution in the lower phase indicates a positive result. **Test for cardiac glycosides** (Keller-Kiliani test): About 1.25mg of each extract was allowed to react with 0.5ml chloroform and mixed carefully. About 0.5ml of concentrated sulfuric acid was then carefully added to form a lower layer. The reddish-brown color at the interface indicates the presence of a steroidal ring, the glycone portion of cardiac glycosides. **Test for flavonoids**: About 7.5mg of each dry extract was dissolved in 0.5ml of ethanol, concentrated HCl, and magnesium turnings. A yellowish coloration indicates the presence of flavonoids.

#### Test for phenolic compounds (Ferric Chloride Test)

The crude extract of the plant material was treated with 3 to 4 drops of ferric chloride solution, or dissolving 5mg of dry extract in 0.5ml of 1% ferric chloride solution. The formation of bluish-black color indicates the presence of phenolic compounds. **Test for saponin** (Frothing test): About 2.5mg of the plant extract was allowed to be reacted with 5ml water and shaken properly in a test tube. Samples showing froth were warmed. Persistent foam formation indicates the presence of saponin. **Test for tannins** (Braymer’s Test): About 2.5mg of each plant extract was boiled in 5ml of water in a test tube and then filtered through Whatman’s no. 1 filter paper. Two to three drops of 0.1% ferric chloride added and read for brownish green or a blue-black precipitate indicating a positive result. **Test for terpenoids** (Salkowski test): About 0.5ml of the chloroform extract of the dried extracts was evaporated to dryness on a water bath and heated with 3ml of concentrated sulfuric acid for 10 minutes on a water bath. The gray color indicates the presence of terpenoids.

### 2.11 Statistical analysis

The triplicate data reading values of inhibition zones in diameter and concentration values (MIC and MBC) were analyzed using Statistical Packages for Social Sciences (SPSS) software, Window version 22 according to CLSI. Each experimental value was expressed in terms of mean (SD) (mean and standard deviation). Significance in the difference between the two groups was tested by Student t-test, assessed by comparing the corresponding p-value of the test. The *P*-values < 0.05 were considered significant for the study.

### 2.12 Ethics approval and consent to participate

The Research Ethics Committee under the Office of Vice President for Research of Madda Walabu University, the only University in Bale Zone with its main campus in Bale-Robe and the Berbere district administrative office bilaterally approved the fieldwork of this study. On the other hand, as the research was joint collaborative research between Madda Walabu and Kotebe Metropolitan Universities, the Research Ethics Committee of Menelik Medical and Health Science College, Kotebe Metropolitan University in Addis Ababa, Ethiopia granted an ethical approval letter for the study to make use of the patient samples collected at its disposal and other two hospitals in Addis Ababa under its supervision (Ref. No.: IRB/KMU/8974/12). The ethically cleared study, from which we extracted samples from the human subjects (urine, throat swab, and blood samples) was entitled "Molecular characterization of multidrug-resistant microbes among antenatal care attending pregnant women in Addis Ababa" and principally carried out by one of the co-authors of this study. His research team underwent all the standard procedures of ethical clearance, where informed written consent was obtained from all study participants prior to study participation. For those participants who were below the legal consenting age (15.5 to 18 years), they provided written assent, and additionally, their parents/guardians/spouse provided written informed consent. Furthermore, the current study followed all the standard procedures of random representative sample selection (simple random sampling) for the inclusion of unbiased patient samples collected from Addis Ababa into the study subjects, after we had had full permissions from Menelik Tertiary Level Hospital, Kotebe Metropolitan University.

## Results

The dried and powdered leaves of *P*. *aethiopica*, *C*. *englerianum*, *E*. *depauperata*, *L*. *adoensis*, *D*. *penninervium*, and root of *C*. *pustulatus* and *R*. *abyssinicus* were extracted with 80% methanol and screened for their antibacterial and antifungal activities against human pathogens. They had shown promising antimicrobial effects on *E*. *coli*, *K*. *pneumoniae*, *S*. *aureus*, *S*. *pyogenes*, *C*. *albicans*, and *T*. *mentagrophytes*.

### 3.1 Antibacterial effect of extracts on multidrug-resistant bacteria

**Fig 1:**
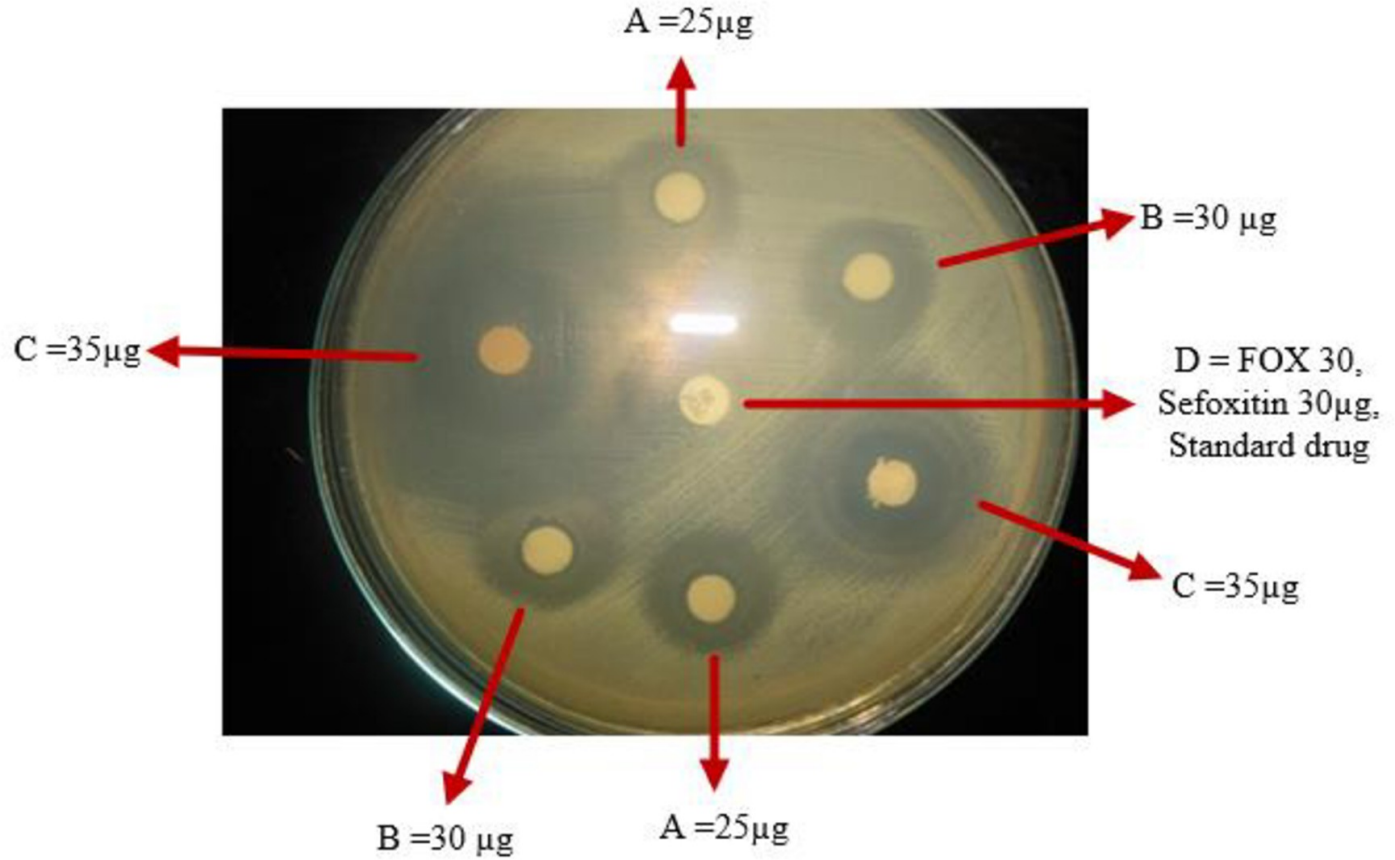
Antibacterial effect of *Cirsium englerianum* on MRSA in different doses.

Almost all extracts showed antibacterial activity against two or more multidrug-resistant and reference strains of human pathogenic bacteria. The extract of *E*. *depauperata*, *R*. *abyssinicus*, and *L*. *adoensis* showed the broadest spectrum of action as they inhibited the growth of *E*. *coli*, *K*. *pneumoniae*, *S*. *aureus*, and *S*. *pyogenes* with a zone of inhibition in diameter ranging from 21- 29mm (Table 1). The methanolic extract of *C*. *englerianum*, *L*. *adoensis*, and *E*. *depauperate* showed inhibition zone in diameter values of 28mm, 27mm, and 26mm on the MRSA, respectively. The novel antimicrobial activity was observed from the methanolic extract of *C*. *englerianum* against the *S*. *pyogenes*. It had an inhibition zone of 29mm against both strains collected from the sample and reference strain. In addition, *S*. *aureus* had a remarkable inhibition zone (Fig 1). The methanolic extract obtained from *E*. *depauperata* revealed appreciable antibacterial activity against MSSA and MRSA with an inhibition zone of 27mm and 26mm, in that order (Table 1).

**Table 1:** Methanolic extracts inhibition zone in diameter on human pathogens on agar disc diffusion method performed at AAU, 2018.

Scientific name	μg/ml	Inhibition zone in diameter (mm)									
		Gram-positive bacteria				Gram-negative bacteria				Fungi	
		MRSA®	MSSAR	*S*.*pyogen*®	*S*.*pyogen*R	*E*.*coli*®	*E*.*coli*R	*K*.*pne®*	*K*.*pneR*	*C*. *aR*	*T*.*rR*
*C*.*englerianum*	30	28(0.8)	28(3.0)	29(1.3)	29(1.5)	22(0.7)	23(0.2)	28(0.8)	28(0.9)	23(0.7)	11(0.3)
	10	17(0.4)	18(0.1)	20(0.9)	19(1.0)	11(0.3)	15(1.4)	18(2.0)	19(0.1)	13(1.1)	-
*E*. *depauperata*	30	26(1.0)	27(2.4)	28(0.9)	28(0.5)	15(0.1)	14(0.4)	27(1.2)	27(0.7)	-	25(0.7)
	10	17(0.7)	17(0.6)	18(2.0)	19(0.3)	-	-	19(0.1)	19(0.7)	-	13(0.9)
*L*. *adoensis*	30	27(1.8)	27(2.4)	26(0.5)	26(1.5)	25(2.1)	24(0.3)	29(0.1)	29(0.7)	15(0.9)	23(0.1)
	10	19(0.1)	18(1.2)	19(1.1)	18(0.1)	10(0.7)	14(0.2)	20(0.6)	20(0.3)	-	11(0.3)
*D*.*penninervium*	30	25(2.0)	25(0.5)	24(0.1)	22(1.0)	27(0.8)	27(0.1)	28(0.7)	29(0.4)	20(0.9)	21(0.4)
	10	16(0.1)	16(0.3)	17(0.4)	17(0.7)	20(0.7)	19(1.0)	18(0.3)	18(0.2)	13(0.3)	9(1.6)
*R*.*abyssinicus*	30	23(0.4)	23(0.6)	27(0.4)	27(1.1)	12(0.8)	12(0.1)	24(0.1)	24(0.6)	22(0.5)	26(0.1)
	10	12(0.8)	13(0.9)	15(1.1)	15(1.0)	-	-	14(1.0)	15(0.7)	10(0.2)	14(0.4)
*C*. *pustulatus*	30	22(0.8)	22(0.8)	24(0.5)	23(0.8)	23(0.4)	18(0.9)	25(1.0)	24(0.4)	18(0.1)	20(0.8)
	10	13(0.2)	12(1.0)	12(0.8)	15(1.2)	15(0.2)	10(0.8)	15(0.9)	14(0.8)	12(0.8)	11(0.1)
*P*.*aethiopica*	30	19(0.1)	19(0.2)	20(0.1)	21(0.1)	-	-	19(0.3)	19(0.4)	-	-
	10	10(0.5)	9(0.2)	12(0.6)	12(0.6)	-	-	12(0.4)	11(0.9)	-	-
Modern drug	-	-	28(0.1)C	NT	NT	-	26(2.1)C	-	32(1.2)c	28(0.3)F	26(1.3)F

The values represent mean (standard deviation), NT = Not tested,— = No inhibition zone, ® = multidrug resistant strains<16mmIZ, R = reference strain (susceptible) > 21mmIZ, C.a = *C*. *albicans*, *T*.*r = T*. *mentagrophytes*, *K*.*pne = K*.*pnemonae*, C = Ciprofloxacin, F = ketoconazole, Where, P < 0.001when compared to cefoxitin treated MRSA. While, #P < 0.05, ##P < 0.01 when compared to modern drug treated K. pneumoniae and P < 0.01 when compared to modern drug treated E. coli *P < 0.05, ¥**P < 0.01, P < 0.05, ¥¥**.

**Fig 2:**
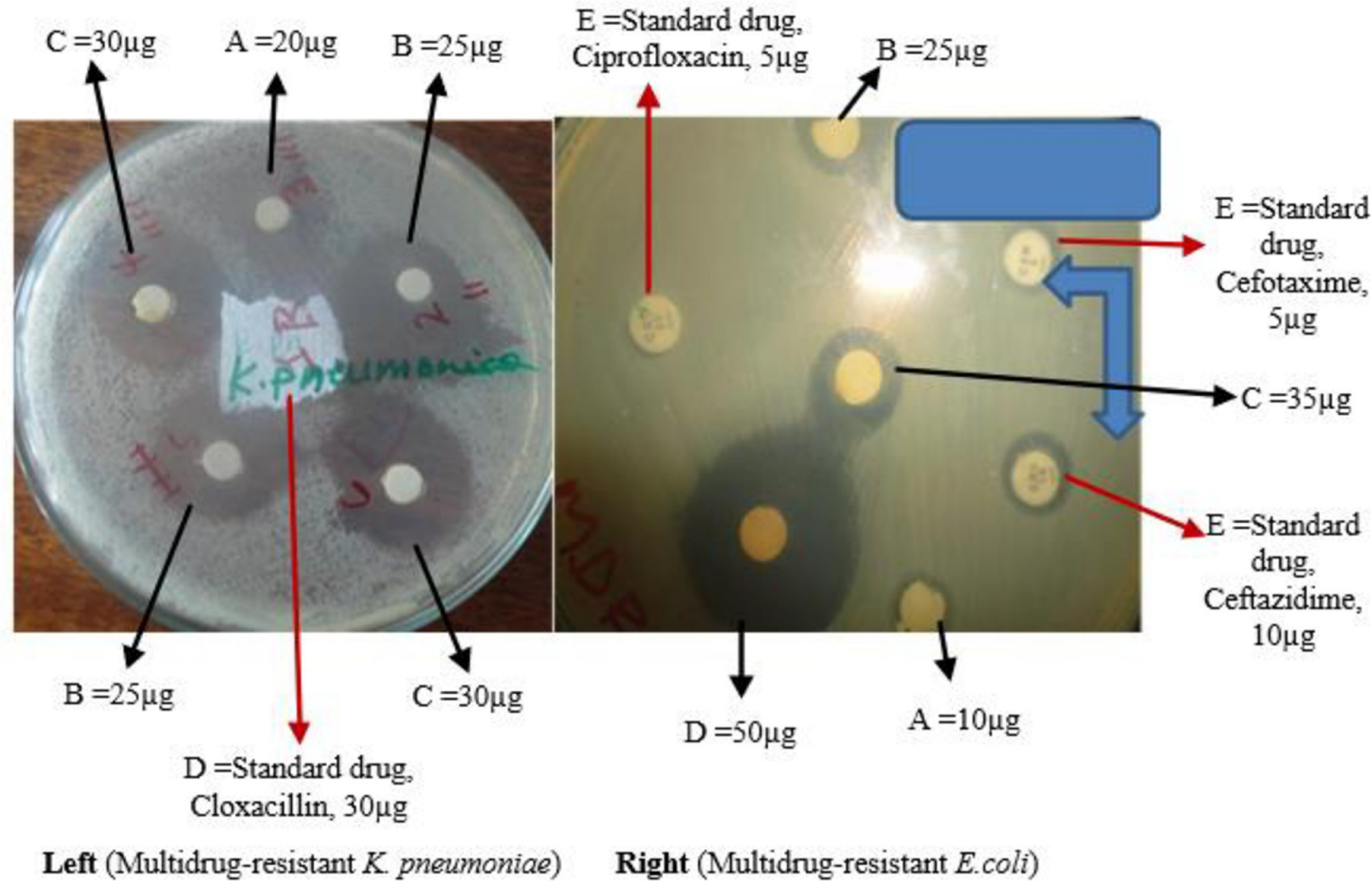
Antibacterial effect of *Cirsium englerianum* on MDR *K*. *pneumoniae* and *E*. *coli*.

On the other hand, MDR gram-negative pathogenic bacteria, *K*. *pneumoniae* strains were the second susceptible bacteria. It had an inhibition zone in diameter ranging from 19mm to 29mm. Similarly, another MDR gram-negative pathogenic bacteria, *E*. *coli* had shown 15mm, 22mm, and 27mm inhibition zone in diameter when the extract obtained from *E*. *depauperata*, *C*. *englerianum*, and *D*. *penninervium* were applied, respectively (Table 1). However, the extract from *P*. *aethiopica* failed to show an observable zone of inhibition against the MDR *E*. *coli* bacteria tested (Fig 2).

The MIC value of plant extracts against the tested Gram-positive bacteria ranged from 1.0 to 64.0μg/ml. The lowest MIC and MBC values were observed in the case of *S*. *pyogenes*, which were the most inactivated bacteria species by most methanolic extracts. Also, these findings divulge remarkable growth inhibition of both MRSA, with MIC and MBC value ranges of 4.0 to 64.0μg/ml (Table 2). The second most inactivated bacteria were clinical isolate MDR and reference strain of *K*. *pneumoniae*, with MIC and MBC value ranges of 2.0 to 64.0 μg/ml and 2.0 to 128.0μg/ml, respectively (Table 2). The minimum bactericidal concentration test was directly proportional to its respective MIC value. The bacteria species inhibited at the lower concentration of the plant extracts also showed no growth in subculture media with relatively lower concentration.

**Table 2:** MIC and MBC/MFC values of the methanolic extract against human pathogens on agar dilution method performed at AAU, 2018.

Scientific name	MIC/MBC MFC	The concentration of extracts in μg/ml on human pathogen bacteria and fungi									
		Gram-positive bacteria				Gram-negative bacteria				Fungi	
		MRSA	MSSA	*S*. *pyogen*®	*S*. *pyogenR*	*E*.*coli*®	*E*.*coli* R	*K*.*pne*®	*K*.*pne*R	C.a	T.r
*C*.*englerianum*	MIC	16.0	16.0	1.0	1.0	64.0	64.0	2.0	2.0	128.0	64.0
	MBC/MFC	32.0	32.0	2.0	2.0	128.0	128.0	2.0	2.0	128.0	128.0
*E*. *depauperata*	MIC	4.0	4.0	4.0	4.0	128.0	128.0	16.0	16.0	NT	64.0
	MBC/MFC	4.0	8.0	8.0	8.0	128.0	128.0	16.0	16.0	NT	128.0
*L*. *adoensis*	MIC	64.0	32.0	16.0	16.0	64.0	64.0	16.0	16.0	384.0	512.0
	MBC/MFC	128.0	128.0	16.0	16.0	64.0	64.0	32.0	32.0	NT	NT
*D*. *penninervium*	MIC	8.0	8.0	8.0	16.0	16.0	16.0	64.0	64.0	128.0	128.0
	MBC/MFC	16.0	16.0	8.0	8.0	16.0	16.0	128.0	128.0	128.0	128.0
*R*. *abyssinicus*	MIC	16.0	16.0	64.0	64.0	128.0	128.0	16.0	16.0	32.0	16.0
	MBC/MFC	32.0	16.0	128.0	128.0	64.0	64.0	32.0	16.0	64.0	32.0
*C*. *pustulatus*	MIC	64.0	64.0	4.0	4.0	32.0	32.0	8.0	8.0	128.0	256.0
	MBC/MFC	64.0	64.0	8.0	8.0	64.0	64.0	16.0	16.0	128.0	NT
*P*. *aethiopica*	MIC	16.0	32.0	8.0	8.0	NT	NT	64.0	64.0	NT	NT
	MBC/MFC	64.0	64.0	16.0	16.0	NT	NT	128.0	128.0	NT	NT
Modern drug		+	0.1C	NT	NT	+	0.1C	+	0.1C	0.3F	0.3F

® = multidrug-resistant strains, R = reference strain (ATCC) for each species, C.a = *C*. *albicans*, *T*.*r = T*. *mentagrophytes*, NT = Not tested, *K*. *pne = K*. *pneumonia*, C = 5μg ciprofloxacin, F = 20μg ketoconazole, S = strains from the sample, + = Growth has been seen.

### 3.2 Antifungal effect of extracts on fungi

This study revealed that the methanolic extracts obtained from *C*. *englerianum*, *L*. *adoensis*, *D*. *penninervium*, *and R*. *abyssinicus* demonstrated remarkable antifungal effects against *C*. *albicans* and *T*. *mentagrophytes* (Table 1). The methanolic extract of *R*. *abyssinicus* showed a promising antifungal effect over others. It had a 26mm inhibition zone in diameter and MIC values ranging from 32 to 64μg/ml. Likewise, extracts of *D*. *penninervium* and *C*. *englerianum* showed inhibitory activity against fungi at MIC/MFC value range of 16 to 512μg/ml, while *L*. *adoensis* was found less effective against human pathogenic fungi in a MIC range of 384 to 512μg/ml. No antifungal activity was observed with extracts of *P*. *aethiopica* against *C*. *albicans* and *T*. *mentagrophytes* (Table 2).

### 3.3 Phytochemical screening from the medicinal plants extract

The tested medicinal plants showed significant variation in the percentage yield with 80% methanol extraction by maceration. The highest yield was observed in *C*. *englerianum* (38%), while the lowest yield was detected in *E*. *depauperata* (22%) (Table 3). In terms of the qualitative phytochemical investigation of the medicinal plants, the medicinal plants extract had different phytochemicals constituents such as saponins, tannins, alkaloids, terpenoids, anthraquinones, phenolic compounds, cardiac glycosides, and flavonoids (Table 4). These bioactive compounds are naturally occurring in most extracts and identified to possess bactericidal or fungicidal properties on the tested human pathogens.

**Table 3:** Ethno-botanical data and percentage yields of medicinal plants tested on selected human pathogens.

Scientific name	Family	Local name	Part used	% yield, mean (SD)	Locally used to treat
*Polysphaeria aethiopica* Verdc.	Rubiaceae	Qarraruu	leaf	36 (2.3)	Toothache, scabies, oral inflammation, TB
*Cirsium englerianum*	Asteraceae	Adaddoo	Leaf	38.1 (1.0)	Gonorrhea, tonsillitis, syphilis, wound amebiasis, malaria, diarrhea
*Euphorbia depauperata*	Euphorbiaceae	Gurii	Bark	22.3 (0.9)	Skin rash, ringworm, bloody diarrhea, gastritis, constipation
*Lippia adoensis*	Verbenaceae	Urgoo	Leaf	27.3 (1.7)	Stomach ache, diarrhea, wound, and cough
*Discopodium Penninervium*	Solanaceae	Maraaro	Leaf	33.9 (0.4)	Eczema, wound, scabies, urine retention
*Cucumis pustulatus*	Cucurbitaceae	Haadhatu	Root	29.1 (2.8)	Cough, TB and chest pain, cold disease, Pneumonia
*Rumex abyssinicus*	Polygonaceae	Dhangagoo	Root	24.0 (0.3)	Gonorrhea, skin diseases, diarrhea, abscesses, ringworm, pain-relieving, diuretic effect, hypertension, anti-cancer, malaria, wound healing

**Table 4:** Preliminary phytochemical screening of some traditional medicinal plants collected from Bale Zone.

Scientific name (parts)	Solvent used for extraction	Major phytochemicals in the crude extract of medicinal plants							
		Saponins	Tannins	Alkaloids	Terpenoids	Anthraquinone	Flavonoids	Phenolic	Cardiac glycoside
*C*.*englerianum*	Methanol	+	++	+++	+	+	+++	+++	++
(fruit)	Aqueous	-	++	++	+	-	+	+++	++
*E*. *depauperata*	Methanol	+++	-	++	+	+	+++	+	+
(Bark)	Aqueous	++	-	+	-	++	++	++	-
*L*. *adoensis*	Methanol	++	+	+++	+	+++	++	+	-
(Leaf)	Aqueous	-	+	+	-	+	++	+	+
*D*.*penninervium*	Methanol	+++	+	+++	+	++	+++	-	++
(Leaf)	Aqueous	+	+++	++	+	++	+	-	+
*C*. *pustulatus*	Methanol	+	++	-	-	++	+	++	+
(Root)	Aqueous	+	_	-	+	+	+	+	++
*R*. *abyssinicus*	Methanol	++	+	+	++	+++	+	++	-
(Root)	Aqueous	+++	+	++	+	++	-	++	+
*P*. *aethiopica*	Methanol	++	+	++	+++	-	++	++	-
(Leaf)	Aqueous	+	-	++	+	++	++	++	+

+++ = Appreciable amount, ++ = Moderate amount, + = Trace amount,— = Not detected

## Discussion

Biomedical science has exploited plants as the potential sources of drugs to prevent and cure human diseases. The World Health Organization has recognized antimicrobial resistance as a global health security threat that requires action across government sectors and society as a whole [4]. It is obvious that the increasing occurrences of MDR microbes ameliorate socioeconomic crises and dwindle public health status worldwide. These MDR microbes compromise the success of many currently accessible and affordable antimicrobials on the shelf, especially in developing countries. It is for these reasons that the search for new antimicrobials from medicinal plants to combat the growing threat from those pathogenic microbes, MDR bacteria, and fungi. This could be demonstrated by almost all tested medicinal plants in this study, where the extracts from the majority of them showed a glimpse of prospective new drug discoveries to cure and control diseases caused by MDR pathogenic microbes (Figs 1 and 2). These findings corroborate previous studies that the therapeutic agents derived from plants are used as an important surrogate, alternative, or complementary treatment of infectious diseases [16–18].

This current study revealed that all species of plants tested had shown antibacterial activity against *S*. *pyogenes*, as this bacterium *was* the most susceptible species of all tested human pathogenic bacteria. Besides, most of the extracts had a remarkable and pronounced inhibitory effect on MRSA, next to *S*. *pyogenes*. Moreover, multidrug-resistant and the reference strains of *K*. *pneumoniae* were found to be the most sensitive gram-negative bacteria by the majority of the tested plant extracts. Implausible, both multidrug-resistant and reference strains of *E*. *coli* were the most unresponsive strain of all tested bacteria species. These findings agree with several research reports [25, 37]. This demonstrates that gram-positive bacteria (*S*. *aureus* and *S*. *pyogenes*) are more sensitive to TMP extracts tested than the gram-negative (*E*. *coli* and *K*. *pneumoniae*). Unlike the current study result on *E*. *coli*, many studies have shown a glimmer of hope as medicinal plants are endowed with many different bioactive compounds that potentially inhibit the growth of this human bacterial pathogen [19]. On the other hand, researchers demonstrated that *E*. *coli* has developed multidrug resistance to many currently available and affordable antibiotics on the market [20, 27]. Thus, genetic makeup to *E*. *coli* to produce multidrug resistance was favorable to impede the antibiotic effectiveness through impeding permeability and enhancing efflux pump [16, 19]. Consequently, searching for antibacterial activity of the secondary metabolites from medicinal plants is a spotlight lately [24, 29]. In this regard, drug discovery from medicinal plants is a near-future hope to overcome socioeconomic problems and long-term negative impacts as a result of multidrug-resistant bacteria, including MRSA, *E*. *coli*, and *K*. *pneumoniae* [25, 37].

Regarding pathogenic fungi of humans in the current study, the presence of bioactive compounds in traditional medicinal plants inhibited the growth *of C*. *albicans* and *T*. *mentagrophytes*. A study conducted elsewhere by *Shumaia Parvin* reported that a selected extract of *C*. *gigantean* had inhibitory potential on all species of human pathogenic bacteria and fungi except *T*. *rubrum*. Another study conducted on ethanolic extract of *P*. *zeylanica* root also reported exhibiting appreciable antimicrobial activities against *V*. *cholerae*, *E*. *coli*, *P*. *aeruginosa*, *Curvularia lunata*, *Colletotrichum corchori*, and *Fusarium equiseti* [21, 22].

Overall, the current study has shown that the human fungal pathogens were relatively tolerant to many of the tested extracts as compared to the human pathogenic bacteria. Also, among the pathogenic fungi, the current findings demonstrated that *C*. *albicans* was less susceptible to the extracts as compared to the cutaneous mycosis causing fungal agent (*T*. *mentagrophytes*). This might be due to the presence of different bioactive entities that potentially inhibit the growth of cutaneous mycosis, like that of the multidrug-resistant bacteria. The difference in extracts efficacy on the growth of *T*. *mentagrophytes* and *C*. *albicans* indicates the presence of antifungal constituents in the crude extracts of each plant. These findings agree with the previous research reports implicating the antifungal activity of medicinal plants through its different secondary metabolites that potentially inhibit the growth of pathogenic fungi affecting human beings [22, 24].

The preliminary findings of the present study on most endemic medicinal plants from Bale Zone show that most plants screened contain flavonoids, anthraquinones, alkaloids, tannin, phenolic, and saponin bioactive compounds (Table 4). The presence of these bioactive compounds recovered from the traditional medicinal plants was found to inhibit the growth of both reference strains and MDR clinical isolate microbes. Other studies had also documented that medicinal plants contain coumarins, flavonoids, phenolics, alkaloids, terpenoids, tannins, and polyacetylenes which have the potential as a bactericidal, bacteriostatic, or fungicidal effect against selected human pathogens [30, 37]. Some other authors proposed that this inhibitory activity of secondary metabolites emanates from the sequential inhibition of the biochemical pathway, inhibition of protein synthesis, and disintegration of the outer membrane [1, 7]. Thus, many researchers argue that medicinal plants have diversified secondary metabolites that help to refute the notorious diseases caused by MDR infectious agents, such as bacteria and fungi [19]. Therefore, a lot has to be done to explore potential innovative new antimicrobials agent discovery from traditional medicinal plants so as treat MDR human pathogen infections.

It would appear that this piece of paper represents the first published work exploring *in vitro* activity against selected MDR pathogens of humans in the study area. Additionally, it reports that the methanolic extract possessed significant antimicrobial activities over the aqueous extracts. However, our study was not accompanied by a toxicity test. Hence, there is a need to conduct the cytotoxicity of the active plant extracts against Vero cell lines to prove the safety of the extracts. Toxicity studies also potentially prove or disprove the scientific dilemma of anticancer activities of such traditional medicinal plants claimed by the local communities in the study area. In addition, it is necessary to further investigate the biological activities, particularly clinical efficacy trial and affordability analyses, related to those plant extracts with positive responses above the minimum threshold as antimicrobials.

## Conclusions

Multidrug-resistant (MDR) pathogens are a growing threat to human health and welfare. The presence of MDR infections, including those of *Staphylococcus aureus*, in both hospitals and communities, is disturbing to healthcare providers due to the difficulty in treating these infections, resulting in longer hospital stays and increased patient morbidity and mortality. Recent estimates suggest up to 55% of the *S*. *aureus* isolates are MDR. Besides, a more even serious threat to humanity is the MDR pathogenic coliform bacteria; *Klebsiella pneumoniae* and *Escherichia coli* posing few therapeutic options for the treatment of infections caused by them. Thus, there needs to be a concerted effort to research and develop new treatment strategies for the management of both methicillin-resistant *S*. *aureus*, *K*. *pneumoniae*, and *E*. *coli* and human pathogenic fungi. To this effect, Bale Zone is the hub of enormous plant biodiversity potential in terms of offering a wide range of traditional medicinal plants, and the powerhouse of natural pharmacy on the Earth; the Harenna Forest and its adnexa territories which extends down to the neighboring Berbere district with a plethora of indigenous florae. The present study on selected medicinal plants is found to demonstrate more antibacterial effects than antifungal activities. Most of the tested traditional medicinal plant extracts have a promising antimicrobials effect on MDR bacteria. The current study seems to unravel further detailed investigations on the plant extracts showing appreciable antimicrobial responses against MDR pathogenic microbes of human; in terms of the (low risk of) toxicity, clinical efficacy trial (*in vivo* experiments), safety tests, and affordability analyses; are necessary to draw reliable conclusions. As the last lap of the journey towards the discovery of new and more efficient antibacterial agents from the extracts is greenlighted by these later indicated tests and utterly essential.

## List of abbreviations

ATCC: American Type Culture Collection used as reference strains for respective bacteria
CLSI: Clinical Laboratory Standard Institute
ESBL: Extended Spectrum Beta Lactamase
MBC: Minimum Bactericidal Concentration
MDR: Multi Drug-Resistant
MHA: Muller Hinton Agar
MHB: Muller Hinton Broth
MRSA: Methicillin-Resistant *Staphylococcus aureus*
MSSA: Methicillin Susceptible *Staphylococcus aureus*
MBC: Minimal Bactericidal Concentration
MIC: Minimal Inhibitory Concentration
TMP: Traditional Medicinal Plants
